# Aortic Root Replacement in Patients With Ventricular Septal Defect

**DOI:** 10.1016/j.atssr.2025.06.025

**Published:** 2025-07-28

**Authors:** Ryota Takahashi, Hiroshi Munakata, Kenji Okada, Taro Hayashi, Tatsuichiro Seto, Hisao Suda, Yutaka Okita

**Affiliations:** 1 Takatsuki General Hospital, Osaka, Japan; 2 Okinawa Southern Medical Center, Okinawa, Japan; 3 Kobe University, Kobe, Japan; 4 Akashi Medical Center, Akashi, Japan; 5 Shinshu University, Matsumoto, Japan; 6 Nagoya City University, Nagoya, Japan

## Abstract

**Background:**

Aortic root replacement in patients with ventricular septal defect (VSD) requires a modified surgical technique.

**Methods:**

Between 2000 and 2022, 12 patients with aortic regurgitation and VSD underwent an operation. Their age at operation was 31.8 (SD 19.9) years. The VSD was patent in 5 patients and spontaneously or surgically closed in 7 patients. The location of the VSD was subarterial in 6 patients, perimembranous in 5, and muscular in 1 patient. The aortic valve was tricuspid in 8 patients, bicuspid in 2, and unicuspid in 2. Eight patients also had annuloaortic ectasia.

**Results:**

The VSDs were closed using a patch in 4 patients and directly closed in 1 patient. The aortic root procedures were valve-sparing reimplantation in 7 patients, root remodeling in 1, basal ring annuloplasty in 1, basal ring annuloplasty with sinutubular junction annuloplasty in 1, and a Ross procedure in 1 patient. Additional cusp repair was required in 9 patients. No early deaths occurred. The postoperative follow-up periods were 5.3 (3.4) years. Two patients died, and 1 underwent aortic valve replacement 4 years postoperatively. Survival was 91.7 (8.0)% at 5 years and 68.8 (20.7)% at 10 years. Freedom from aortic valve reoperation was 88.9 (10.5)% at 10 years.

**Conclusions:**

Valve-sparing root reimplantation in patients with annuloaortic ectasia and VSD may require a special first row suture line. Patients with prolapsed cusps may require resuspension or cusp extension. The Ross operation can be an alternative for patients with severely deformed aortic cusps.

## Visual Abstract

**Figure:**
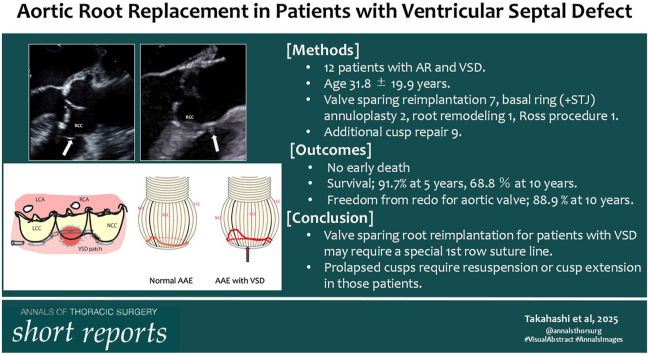

**In Short**

▪ VSRR for patients with AAE and VSD may require a special suture line in the first row.
▪ Prolapsed cusps may require a cusp extension technique.
▪ The Ross operation can be an alternative for patients with severely deformed aortic valve cusps.

Aortic valve cusp prolapse and aortic regurgitation (AR) are common findings associated with ventricular septal defect (VSD) in pediatric patients, including adolescent patients.^1^ A valve-sparing operation has been the first choice to treat these patients. However, several anatomic characteristics may preclude the use of an aortic valve repair technique. Aortic root replacement in patients with VSD sometimes requires modified surgical techniques. We analyzed surgical experiences of AR treatment in patients with VSD or in patients who had operative or natural closure of VSD.

## Patients and Methods

**Figure 1:**
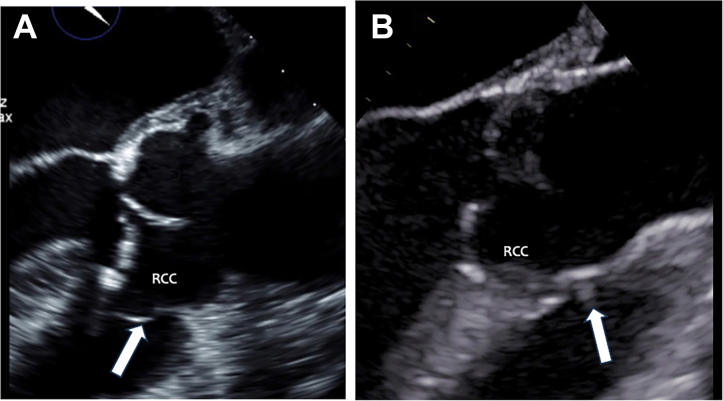
(A) Aortic regurgitation (AR) with a patent perimembranous ventricular septal defect (VSD; arrow). (B) AR with a closed subarterial VSD (arrow). (RCC, right coronary cusp.)

Between 2000 and 2022, 12 patients who had AR and VSD or previously closed VSD underwent surgery. Their age at operation was 31.8 (SD 19.9) years, and their median age was 25 years, ranging from 13 to 74 years. The male-to-female ratio was 11:1. VSD was patent in 5 patients (Figure 1A), spontaneously closed in 2 patients (Figure 1B), and surgically closed in 5 patients (Table 1). The location of VSD was subarterial in 6 patients, perimembranous in 5, and muscular outlet in 1 patient. Their previous operations except for VSD closure were aortic valvotomy, correction of supraaortic valve stenosis, correction of transposition of the great arteries, and double-switch operation for corrected transposition of the great arteries. Anatomy of the aortic valve was tricuspid in 8 patients, bicuspid in 2, and unicuspid in 2 patients; 10 patients had AR, and 1 had aortic valve stenosis. Eight patients also had annuloaortic ectasia (AAE). Mitral valve regurgitation was found in 2 patients, and coarctation of the aorta was noted in 1 patient. Because this study was retrospective and the Japan Cardiovascular Surgery Database Organization obtained informed consent from all surgical patients, consent from each patient was waived. This study was approved by the Institutional Review Board of the Takatsuki General Hospital (2025-10).

**Table 1:** Patient Characteristics

Variables	Years, Ratio, or Number
Study period	2000-2022
Male-to-female ratio	11:1
Age at operation, y	Average 31.8 (19.9), median 25 (range, 13-74)
Ventricular septal defect	
Patent	5
Spontaneously closed	2
Surgically closed	5
Location	
Subarterial	6
Perimembranous	5
Muscular outlet	1
Previous operation	4
Aortic valvotomy	
Patch enlargement of supravalvular aortic stenosis	
Arterial switch for TGA	
Double-switch operation for cTGA	
Lesions	
Bicuspid aortic valve	2
Unicuspid aortic valve	2
Annuloaortic ectasia	8
Aortic regurgitation	10
Aortic stenosis	1
Associated lesions	
Mitral regurgitation	2
Coarctation of the aorta	1

cTGA, corrected TGA; TGA, transposition of the great arteries.

## Results

The VSDs were closed using a patch in 3 patients with the subarterial type and in 1 patient with muscular outlet VSD; the VSD was closed directly in 1 patient with the perimembranous type (Table 2). The aortic root procedures performed were valve-sparing reimplantation in 7 patients, root remodeling in 1, basal ring annuloplasty in 1, basal ring annuloplasty with sinutubular junction annuloplasty in 1, and a Ross procedure in 1 patient. Additional cusp repair was required in 9 patients; repair was completed using a polytetrafluoroethylene suture (Gore-Tex; W.L. Gore & Associates) resuspension technique in 4 patients, central plication in 3, pericardial patch augmentation in 2, and bicuspidization of a unicuspid aortic valve in 1 patient. Other procedures performed were mitral valve repair in 3 patients, reconstruction of the right pulmonary artery using a Gore-Tex tube in 1 patient, and hemiarch replacement in 1 patient.

**Table 2:** Operation

Procedures or Times	Number of Procedures, Number of Patients, or Minutes
Aortic valve procedures	
VSRR reimplantation	7
VSRR remodeling	1
Basal ring annuloplasty	1
Basal ring with STJ annuloplasty	1
Ross operation	1
VSD closure	
Patch	4 (3 perimembranous, 1 muscular outlet)
Direct	1 (perimembranous)
Aortic cusp repair	9 patients
Resuspension	4 cusps, 4 patients
Central plication	4 cusps, 3 patients
Patch augmentation	2 cusps, 2 patients
Bicuspidization of UAV	1 patient
Resuspension failure–Ross operation	1 patient
Other procedures	
Mitral valve repair	3
Tricuspid annuloplasty	1
PA reconstruction	2
Hemiarch replacement	1
Times, min	
CPB time	Mean 231 (45.3), median 235 (range, 153-295)
Cardiac ischemic time	Mean 183 (40.1), median 178 (range, 123-255)

CPB, cardiopulmonary bypass; PA, pulmonary artery; STJ, sinutubular junction; UAV, unicuspid aortic valve; VSD, ventricular septal defect; VSRR, valve-sparing root replacement.

**Figure 2:**
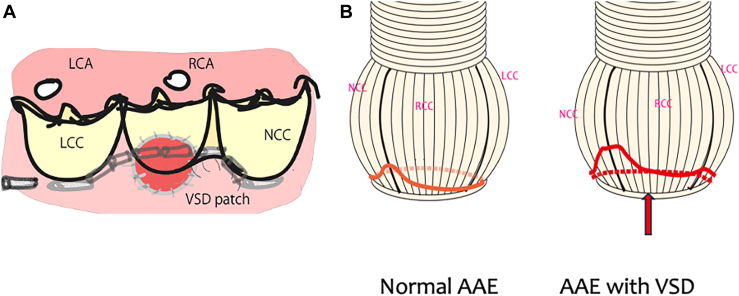
(A) Ventricular septal defect (VSD) patch impeding deep dissection of the aortic root during the aortic root reimplantation procedure, (B) First row suture line (red line) required to deviate cranially from the normal reimplantation technique (arrow). (AAE, annuloaortic ectasia; LCA, left coronary artery; LCC, left coronary cusp; NCC, noncoronary cusp; RCA, right coronary artery; RCC, right coronary cusp.)

Deep dissection of the aortic root during the aortic root reimplantation procedure was impeded by the presence of a VSD patch (Figure 2A). Particularly in patients with a subarterial defect, the first row suture line in the reimplantation technique had to cross the VSD patch. The first row suture line (Figure 2B, red line) was required to deviate cranially from the normal reimplantation method (Figure 2B, arrow). The Video shows a 66-year-old male patient with severe AR and subarterial VSD. The subarterial VSD was closed with a bovine pericardial patch and 4-0 pledgeted sutures through a pulmonary arteriotomy. Dissection of the aortic root was difficult in the right coronary artery area because of the presence of the VSD patch. A small tear was found in the right coronary cusp near the commissure. Aortic root reimplantation was applied using a 26-mm Valsalva polyester (Dacron; INVISTA) graft. First row sutures were deviated superiorly around the right coronary sinus. Aortic cusp repair was performed using an autologous pericardial patch (Video). The mean cardiopulmonary bypass time was 231 (SD 45.3) minutes, and the median time was 235 minutes, ranging from 153 to 295 minutes. The mean cardiac ischemic time was 183 (40.1) minutes, and the median time was 178 minutes, ranging from 123 to 255 minutes.

No early deaths occurred, and all patients were discharged within 1 month. The postoperative follow-up periods were 5.3 ± 3.4 years, up to 11.1 years. Two patients died of unknown causes 3 and 8 years postoperatively. One patient underwent an aortic valve replacement reoperation 4 years after the initial operation because of recurrent AR. Survival was 91.7 (8.0)% at 5 years and 68.8 (20.7)% at 10 years. Freedom from reoperation for the aortic valve was 88.9 (10.5)% at 10 years.

## Comment

Valve-sparing root replacement (VSRR) has shown promising results in patients with congenital heart defects and aortic root dilatation.^2^ Studies report good long-term survival and aortic valve function in most patients. The reimplantation technique (David procedure) is commonly used, with some centers also using remodeling techniques. However, valve degeneration remains a concern, and some patients require subsequent aortic valve replacement.^3^ An aortic valve-sparing operation is a preferred method in younger patients with AAE, and the David reimplantation technique has prevailed because of a superior long-term freedom from AR in these subsets of patients.^2^ Aortic cusps sometimes prolapse into the VSD, with the left side of the right coronary cusp through the subarterial VSD and, in patients with a perimembranous VSD, noncoronary cusp, or commissure of the right coronary cusp and noncoronary cusp prolapse.^4^ VSRR can be extended to this setting to ameliorate the consequent aortic cusp derangement and commissural destabilization.

In younger pediatric patients, simple patch closure of the VSD may correct aortic cusp prolapse,^5^ but the aortic cusp should be repaired in grown children or in adult patients because long-standing cusp prolapse may cause secondary degeneration, such as cusp thickening, shrinkage, or even tearing.^6^ The cusp plication technique is widely used to correct the prolapsing cusps, and most cusps are stretched and have redundant free margin length. We used the free margin resuspension technique using polytetrafluoroethylene sutures and reported that free margin resuspension is more promising than central plication because the resuspension technique provided an even mechanical stress distribution along the free margin of the aortic cusp.^7^ Nevertheless, the cusp repair technique may become more complicated in these patients, and pericardial patch augmentation is sometimes required. The long-term outcomes of the aortic cusps, where autologous pericardial patch was applied, have been suboptimal.^8^

Deep aortic root dissection to the horizontal plane of the basal ring, by placing the first row sutures horizontally, and using the cylindrical Dacron graft are thought to be key to achieve aortic valve competency during the reimplantation procedure. However, the presence of VSD or VSD patches precludes dissection of the aortic root from the right ventricular muscle around the right coronary sinus. The same phenomenon occurs in patients with the right coronary sinus buried in the right ventricle, “sinking sinus.” Usually, the first row suture line at the right coronary cusp was placed 5 to 10 mm above the horizontal plane, depending on the depth of dissection. The suture could be placed through the VSD patch.^9^

The aortic root remodeling technique of Yacoub and colleagues^10^ can be an alternative to the David reimplantation technique in these patients because deep root dissection is not required in the remodeling method. However, an intrinsic drawback of basal ring instability of the original remodeling technique encouraged surgeons to add annuloplasty in the basal ring, particularly in young patients or patients with connective tissue disease. In older patients with less dilatation of the basal ring, the remodeling technique can be a useful alternative. Another alternative is the Ross procedure, but the VSD patch or fibrous scar tissue of the VSD may preclude harvesting a perfect autograft.

### Study Limitations

This retrospective study was based on a very small number of patients and on a single surgeon’s experience.

### Conclusion

Twelve patients with AR after repair of VSD or with VSD underwent an aortic valve-sparing operation or a Ross procedure. VSRR for patients with AAE and VSD or with a previously closed VSD may require a special first row suture line. Patients with a prolapsed right coronary cusp may require resuspension or cusp extension. The Ross operation can be an alternative for patients with severely deformed aortic cusps.

## Supplementary Data

Video The patient's head is on the left. The patient was a 66-year-old male presenting with heart failure. He was diagnosed as having severe aortic regurgitation (AR) with annuloaortic ectasia and subarterial ventricular septal defect (VSD). After a longitudinal pulmonary arteriotomy, a 15 × 20 mm subarterial VSD was found. The right coronary cusp (RCC) of the aortic valve was herniated in the VSD. The defect was closed with a bovine pericardial patch using seven 4-0 polyester buttress sutures. After aortotomy, the RCC was severely deformed and there was a defect of the cusp tissue near the left commissure. The diameter of the basal ring, Valsalva sinus, and sinutubular junction was 25 mm, 37 mm, and 32 mm, respectively. Dissection of the aortic root around the right coronary sinus was difficult because of the presence of VSD. Aortic valve sparing root reimplantation was performed using a 26 mm Dacron Valsalva graft. The first row suture line to the Dacron graft was deviated 5 mm upwards in the right coronary sinus. A small piece of the bovine pericardial patch was applied to a tissue defect of the RCC. Cardiopulmonary bypass time was 247 minutes and cardiac ischemia time was 189 minutes.
